# Obesity and Tolerance to Neoadjuvant Chemotherapy in Breast Cancer: A Retrospective Cohort Study

**DOI:** 10.3390/cancers18060889

**Published:** 2026-03-10

**Authors:** Madalena Silveira Machado, Madalena P. Santos, Catarina Relvas, Margarida Quinto Pereira, Mafalda Sousa, Eugénia Santos, Bernardo Alves Pereira, Joana Parreira, Susana Esteves, Paula Ravasco, Fátima Vaz, Hugo Nunes

**Affiliations:** 1 Instituto Português de Oncologia de Lisboa Dr. Francisco Gentil, R. Prof. Lima Basto, 1099-023 Lisbon, Portugal; mpsantos@ipolisboa.min-saude.pt (M.P.S.); crelvas@ipolisboa.min-saude.pt (C.R.); msqpereira@ipolisboa.min-saude.pt (M.Q.P.); mscsousa@ipolisboa.min-saude.pt (M.S.); esilva@ipolisboa.min-saude.pt (E.S.); bapereira@ipolisboa.min-saude.pt (B.A.P.); mparreira@ipolisboa.min-saude.pt (J.P.); sesteves@ipolisboa.min-saude.pt (S.E.); fvaz@ipolisboa.min-saude.pt (F.V.); hnunes@ipolisboa.min-saude.pt (H.N.); 2 Faculty of Medicine, Center for Interdisciplinary Research in Health (CIIS), Universidade Católica Portuguesa, 2635-631 Sintra, Portugal; pravasco@ucp.pt

**Keywords:** obesity, overweight, breast cancer, neoadjuvant chemotherapy, toxicity, peripheral neuropathy

## Abstract

Background: Obesity is increasingly prevalent and has been associated with poorer outcomes and higher toxicity in breast cancer treatment. Its impact on tolerance to neoadjuvant chemotherapy (NAC) remains understudied. Objectives: This study aimed to determine the prevalence of overweight and obesity among patients with high-risk early-stage breast cancer (BC) treated with NAC. It evaluated the association between obesity and clinically significant chemotherapy-related toxicities. Pathological complete response (pCR), progression-free survival (PFS), and overall survival (OS) were explored. Methods: This is a retrospective cohort study on stage I–III BC assigned for NAC between January 2020 and December 2022. Patients were categorized by body mass index. Patients with obesity were compared with an age-matched non-obese cohort (1:1). The toxicities evaluated were hospitalization, dose reduction, treatment delay, or treatment discontinuation. Multivariable logistic regression models were performed, adjusting for age, comorbidity burden, and carboplatin use. Results: Of 487 eligible patients, 31.2% were overweight and 27.7% were obese. Peripheral neuropathy was more frequent and severe in obese patients (63.7% vs. 36.3%, *p* < 0.001). In multivariable analysis, obesity was independently associated with increased odds of global toxicity (OR = 1.83; 95% CI: 1.08–3.15; *p* = 0.027) and treatment discontinuation (OR = 2.30; 95% CI: 1.18–4.59; *p* = 0.016). Conclusions: Obesity is highly prevalent among high-risk early BC patients. It is independently associated with increased treatment-related toxicity, particularly discontinuation. These findings highlight the need for closer toxicity monitoring in patients with obesity. Reliance on BMI alone may be insufficient, underscoring the potential value of incorporating body composition assessment into clinical practice.

## Simple Summary

Obesity is becoming increasingly common and may influence chemotherapy tolerance and survival outcomes. However, its effect on tolerance to neoadjuvant chemotherapy in real-world practice is not fully understood. This study assessed the prevalence of overweight and obesity among patients with high-risk early breast cancer receiving chemotherapy before surgery and whether obesity is associated with a higher risk of treatment-related side effects. We also explored whether obesity is associated with treatment response and survival outcomes. By better understanding the relationship between obesity and treatment tolerance, these findings may help clinicians identify patients at greater risk of complications and tailor supportive care, as well as treatment planning.

## 1. Introduction

According to official 2022 data from the National Institute of Statistics, more than half of the adult resident population in Portugal had excess body weight: 37.3% are classified as overweight and 15.9% as obese. Obesity prevalence did not differ substantially between men and women, and was particularly higher among adults aged 45 years or older [1].

Globally, it is estimated that in 2021, a high body mass index (BMI) was responsible for 3.7 million deaths from cardiovascular disease, diabetes, cancer, neurological diseases, chronic respiratory diseases, and digestive system diseases [2]. It is estimated that around 4 to 8% of cancer cases are attributed to overweight [3].

Obesity confers an increased risk of breast cancer in postmenopausal women [3,4]. In premenopausal women, the association appears to differ. A meta-analysis including 1.9 million women concluded that overweight in premenopausal women is associated with a lower risk of estrogen receptor-positive breast cancer and an increased risk of estrogen receptor-negative breast cancer [5].

The biological mechanisms linking adiposity and cancer are not yet fully established. Excess body fat promotes changes in endogenous hormone metabolism, chronic inflammation, and insulin resistance or hyperinsulinemia, which may increase cancer risk [6].

Evidence suggests that obesity may be associated with a higher incidence of treatment-related toxicities, such as chemotherapy-induced peripheral neuropathy, cardiotoxicity, skin reactions, and lymphedema after surgery or radiotherapy [6]. In clinical practice, patients with obesity are more likely to undergo dose reductions or treatment delays [7]. However, guidelines do not recommend dose capping at initial chemotherapy prescription, due to concerns regarding compromised treatment efficacy [8].

The impact of obesity on pathological response to neoadjuvant chemotherapy remains controversial. A large meta-analysis including 18,702 patients showed lower rates of pathological complete response in overweight or obese women compared with those of normal weight or underweight. This suggests a potential adverse effect of higher BMI on treatment efficacy [9]. Systematic reviews and meta-analyses further indicate that elevated BMI before, at, or after diagnosis is associated with worse prognosis, including higher risk of recurrence and increased all-cause and breast cancer-specific mortality compared with normal-weight survivors [10].

The increased toxicity observed in patients with obesity may be related to a higher prevalence of comorbidities, such as diabetes, hypertension, and cardiovascular disease, as well as alterations in drug pharmacokinetics affecting drug distribution, metabolism, and elimination [11,12,13,14].

Poorer prognosis in women with obesity may reflect delayed diagnosis, more advanced disease at presentation, or chemotherapy dose reductions [15]. Additionally, adipose tissue-derived leptin and pro-inflammatory cytokines may directly promote tumor proliferation [16], while chronic obesity-related inflammation may induce immunosuppression, impairing anti-tumor immune responses [17].

This study aims to evaluate the prevalence of obesity and overweight among early-stage high-risk breast cancer patients treated with neoadjuvant chemotherapy, and to examine the association between obesity and clinically significant chemotherapy-related toxicities, as well as pathological complete response and survival outcomes.

The prespecified hypothesis of this study was that obesity is associated with increased incidence of significant toxicities during neoadjuvant chemotherapy, particularly peripheral neuropathy.

Ultimately, this study contributes further to the characterization of patients with obesity, and it underscores the potential importance of weight management strategies to prevent toxicities and improve quality of life for women diagnosed with breast cancer.

## 2. Materials and Methods

### 2.1. Setting and Eligibility Criteria

We identified all women with primary diagnosis of breast cancer (BC) who were discussed at the tumor board and referred for an oncology consultation at our center between January 2020 and December 2022. Patients assigned to neoadjuvant chemotherapy (NAC) were screened for eligibility.

Only patients with confirmed stage I–III breast cancer were eligible for inclusion. Exclusion criteria included oligometastatic (stage IV) disease treated with NAC, prior history of invasive malignancy (with the exception of non-melanoma skin cancer and in situ carcinomas), treatment primarily at other institutions, and pregnancy at the time of diagnosis.

Chemotherapy doses were calculated using actual body surface area based on body weight and height, in accordance with clinical practice guidelines. No empirical dose capping was implemented; dose modifications were made only when clinically indicated.

Data collection occurred between 14 and 30 November 2025. Relevant clinical, pathological, and imaging data were extracted anonymously from electronic medical records.

Patients were categorized according to BMI, calculated from height and weight recorded at the first oncology appointment. Patients with obesity and a comparator group of non-obese patients were included for analysis of the primary and secondary endpoints.

The non-obese comparator group was randomly selected prior to outcome assessment. Selection was performed by an investigator blinded to outcome variables, with access restricted to age and BMI subgroup information. Age-stratified frequency matching (<50, 50–70, and >70 years) was applied, with 1:1 distribution relative to patients with obesity within each stratum. Age was selected as matching variable due to well-established association with both obesity prevalence and risk of toxicity during NAC, thereby mitigating age-related confounding.

Menopausal status, comorbidities, and BMI were obtained from the electronic medical records at the first oncology visit. Histologic subtype was extracted from pathology reports and classified into three categories: hormone receptor-positive (HR+) if estrogen receptor expression was ≥10% when assessed by immunohistochemistry; HER2-positive (HER2+) if immunohistochemistry was 3+ or 2+ with confirmation by fluorescence in situ hybridization (FISH); and triple-negative breast cancer (TNBC) if HR expression was <10% and HER2 was negative.

Clinical staging was determined from multidisciplinary tumor board records. The clinical stage at diagnosis and pathological stage at the time of surgery were based on the 8th edition of the American Joint Committee on Cancer Staging Criteria (AJCC). Information on germline genetic testing was retrieved from electronic medical records when available.

For the analysis of obesity as an independent risk factor for treatment-related toxicities, potential confounders included the following:

- Age group (<50, 50–70, >70 years).
- Comorbidity burden (number of comorbidities of interest: hypertension, diabetes, dyslipidemia, chronic renal disease, cerebrovascular accident, and myocardial infarction) categorized as 0, 1, and ≥2.
- Treatment regimen (carboplatin-containing vs. non-carboplatin).

### 2.2. Outcomes

#### 2.2.1. Primary Endpoints

This study’s primary endpoints were to determine the prevalence of obesity and overweight among patients with early-stage BC treated with NAC; to examine the association between obesity and clinically significant toxicity, both as a composite endpoint and by its individual components; and to assess the incidence and severity of peripheral neuropathy.

Obesity was defined as BMI ≥ 30 kg/m^2^, and overweight as BMI between 25 and 29.9 kg/m^2^.

Clinically significant toxicities were defined as a composite endpoint including adverse events resulting in treatment delay, hospitalization, dose reduction, or treatment discontinuation during the entire course of NAC. Only adverse events leading to modification of the planned chemotherapy regimen were recorded. Events were classified as toxicity-related when explicitly documented or clearly supported by contemporaneous clinical documentation indicating a treatment-related adverse event. Treatment delays related to toxicity were counted regardless of duration. The composite endpoint was designed to capture the overall impact of treatment-related toxicity on chemotherapy delivery and dose intensity. For this endpoint, patients were counted once if at least one qualifying event occurred during the NAC treatment course, regardless of the number or type of events experienced. Because these events differ in their clinical implications, individual components (treatment delay, hospitalization, dose reduction, and treatment discontinuation) were also analyzed separately to provide a more detailed characterization of clinically meaningful toxicity patterns.

Peripheral neuropathy was evaluated as a specific toxicity regardless of its impact on treatment modification and was graded according to the Common Terminology Criteria for Adverse Events (CTCAE), version 6.0.

#### 2.2.2. Secondary Endpoints

Pathologic complete response (pCR), progression-free survival (PFS), and overall survival (OS) were determined.

Pathologic complete response was defined as the absence of invasive carcinoma in the surgical specimen. Patients with disease progression or death during the neoadjuvant period were considered non-pCR. PFS was defined as the time from initiation of NAC to local progression, local recurrence, distant recurrence, or death from any cause. Progression or recurrence was determined based on clinical or radiologic evidence of new or worsening lesions, and the event date was defined as the earliest documentation in the medical record or the first imaging confirming recurrence. OS was defined as the time from diagnosis to death from any cause or last follow-up. OS was calculated from diagnosis to reflect the overall disease trajectory, whereas PFS was calculated from initiation of NAC to specifically evaluate outcomes related to systemic treatment exposure.

### 2.3. Sample Size

Sample size was estimated using a power-based approach for unconditional logistic regression to detect an association between obesity (yes/no) and treatment-related toxicity. Calculations were performed with SSizeLogisticBin (powerMediation) [18], using a two-sided α = 0.05 and 80% power, and assuming a 50% exposure to take into account the 1:1 frequency matching. Scenarios with baseline toxicity risk of 20–40% and absolute risk increase of 10–25% (OR ranging from 1.5 to 3.3) were explored [12,14,19]. Required sample size ranged from 108 to 775; a total sample of approximately 270 patients provides adequate power to detect moderate-to-large effects (OR ≥ 2.25).

### 2.4. Quantitative Variables

Age was analyzed as a continuous variable for descriptive statistics but was categorized into pre-specified age groups (<50, 50–70, and >70 years) for inferential analyses. The age categories were chosen based on clinical considerations, as modifications in chemotherapeutic regimens and toxicity risk profiles often vary according to age. BMI was treated as a continuous variable for descriptive analyses but was categorized into standard World Health Organization cutoffs (underweight, BMI < 18.5 kg/m^2^; normal weight, BMI 18.5–24.9 kg/m^2^; overweight, BMI 25–29.9 kg/m^2^; and obese, BMI ≥ 30 kg/m^2^) for stratified and multivariable analyses to facilitate interpretation and clinical relevance.

### 2.5. Statistical Analyses

Baseline characteristics were summarized using descriptive statistics. Categorical variables were reported as absolute and relative frequencies, and group comparisons were conducted using the chi-square test or Fisher’s exact test when appropriate. Missing data were minimal, and no imputation was performed. The association between obesity (yes/no) and chemotherapy-related toxicity outcomes was evaluated using unconditional multivariable logistic regression models. Separate models were fitted to each binary endpoint: global toxicity, dose reductions, treatment discontinuation, dose delay, and hospitalization due to treatment toxicity. All models included, a priori, the following covariates based on clinical relevance: age group (<50, 50–70, and >70 years), comorbidity burden (0, 1, and ≥2), and use of carboplatin (yes/no). Odds ratios (OR) with 95% confidence intervals (95% CI) were reported. Model adequacy was assessed for all fitted multivariable logistic regression models using standard diagnostic procedures. Discrimination was evaluated using the area-under-the-receiver operating characteristics curve (AUC), which exceeded 0.65 in all analyses. Calibration was examined using the Hosmer–Lemeshow goodness-of-fit test, with no evidence of lack of fit (*p* > 0.05). Influence diagnostics (Cook’s distance, leverage, and DFBETA) did not identify influential observations, and no complete or quasi-complete separation was observed. Multicollinearity was assessed using variance inflation factors (VIFs) and generalized variance inflation factors (GVIFs), with all adjusted values below 2. These diagnostics support the estimation of adjusted associations between obesity and toxicity to neoadjuvant chemotherapy, controlling for age, comorbidities, and carboplatin-based treatment.

Follow-up time was estimated using the reverse Kaplan–Meier method. Survival functions for OS and PFS were estimated using the Kaplan–Meier method for the overall cohort and stratified by obesity status.

All tests were two-sided, and a *p*-value < 0.05 was considered statistically significant. Analyses were conducted in R (R Foundation for Statistical Computing).

## 3. Results

### 3.1. Descriptive Data

**Figure 1:**
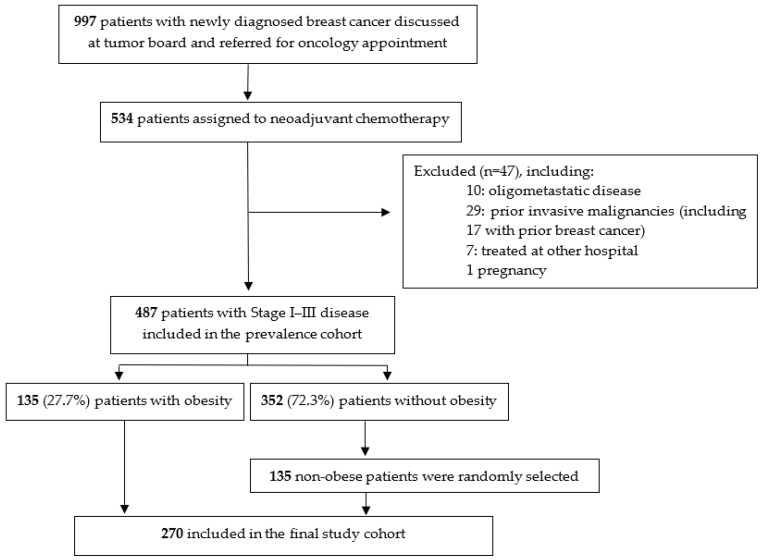
Flowchart of the study cohort.

A total of 487 patients fulfilled the inclusion criteria. Figure 1 shows the flow of participants through the study, including exclusions and final numbers analyzed. Overall, 152 patients (31.2%) were classified as overweight and 135 (27.7%) as obese. Table 1 shows the distribution of patients by age group and BMI category. One patient was male. The median age in the overall cohort was 52 years (range 20–82), and 247 patients (50.7%) were postmenopausal. The median BMI was 26.3 kg/m^2^ (range 16.8–53.5).

Among patients with obesity (PWOs), the median age was 55 years; 45 (33.3%) were younger than 50 years, 75 (55.5%) were aged 50–70 years, and 15 (11.1%) were older than 70 years. Among non-obese patients (NOPs), the median age of was 53 years, with the same age-group distribution. Median BMI was 32.6 kg/m^2^ in PWOs and 24.1 kg/m^2^ in NOPs. Regarding menopausal status, 60% of PWOs and 56.3% of NOPs were postmenopausal.

Patients with obesity had a higher prevalence of comorbidities than NOPs (63.7% vs. 34.1%, *p* < 0.001), including hypertension (51.9% vs. 24.4%, *p* < 0.001) and dyslipidemia (30.4% vs. 13.3%, *p* < 0.01). No significant differences were observed for diabetes (*p* = 0.06), chronic kidney disease (*p* = 1.00), stroke (*p* = 1.00), or myocardial infarction (*p* = 0.65). Patients with obesity showed a higher proportion of HR-positive/HER2-negative tumors (50.4% vs. 39.3%); however, this difference was not statistically significant (*p* = 0.087). Clinical stage and treatment regimens were similar between PWOs and NOPs, as shown in Table 2. Germline genetic testing was less frequently performed in patients with obesity.

### 3.2. Toxicity

Treatment delays, dose reductions, hospitalizations, and treatment discontinuations due to adverse events occurred in both groups, with a higher overall rate in the PWO group (60% vs. 45.2%). Delays were mainly attributable to infections and hematologic toxicities. Dose reductions were most often related to peripheral neuropathy, which was also the leading cause of treatment discontinuation in both groups. Deterioration in performance status also contributed to discontinuation (PWO n = 6, 20.7% vs. NOP n = 1, 5.3%). Death during NAC occurred in two PWOs and three NOPs, while disease progression led to discontinuation in one patient in each group. Hospitalizations occurred more frequently in PWOs, predominantly due to febrile neutropenia. The distribution of specific toxicities is detailed in Table 3.

### 3.3. Peripheral Neuropathy

Peripheral neuropathy occurred in 63.7% (n = 86) of PWOs and 36.3% (n = 49) of NOPs (*p* < 0.001). The distribution by grade was as follows: Grade 1—49 PWOs and 30 NOPs; Grade 2—28 PWOs and 18 NOPs; and Grade 3—9 PWOs and 1 NOPs.

### 3.4. Multivariable Analysis

Obesity was independently associated with a higher risk of global clinically significant toxicity (OR = 1.83; 95% CI: 1.08–3.15; *p* = 0.027) after adjustment for age, comorbidities, and carboplatin use. Among the covariates, advanced age (>70 years) was strongly associated with toxicity (OR = 9.84; 95% CI: 3.18–37.83; *p* < 0.001), whereas age 50–70 years showed a non-significant trend compared with age under 50 years. Comorbidity burden was not independently associated with toxicity. Carboplatin-containing regimens were associated with increased toxicity (OR = 2.33; 95% CI: 1.16–4.84; *p* = 0.020; Table 4).

When outcomes were analyzed separately, obesity was not associated with dose reduction (OR = 1.03; 95% CI: 0.58–1.83; *p* = 0.923), and age > 70 years was the only independent predictor (OR = 8.21; 95% CI: 3.02–23.88; *p* < 0.001; Table 5). Only carboplatin use was strongly associated with treatment delays (OR = 5.73; 95% CI: 2.69–12.46; *p* < 0.001; Table 6), and obesity, age, and comorbidity group were not significantly associated with delays. For treatment discontinuation, obesity was independently associated with increased risk (OR = 2.30; 95% CI: 1.18–4.59; *p* = 0.016), as was age > 70 years (OR = 7.09, 95% CI: 2.34–22.51; *p* < 0.001), while the 50–70-year age group again showed a non-significant trend (Table 7). For hospitalization, age > 70 years was the only factor significantly associated with increased risk (OR = 5.61; 95% CI: 1.11–33.22; *p* = 0.041), whereas obesity did not emerge as an independent predictor (Table 8).

### 3.5. Pathologic Complete Response

Pathologic complete response occurred in 25.2% of PWOs (n = 34) and 29.6% of NOPs (n = 40).

### 3.6. Survival Outcomes

**Figure 2:**
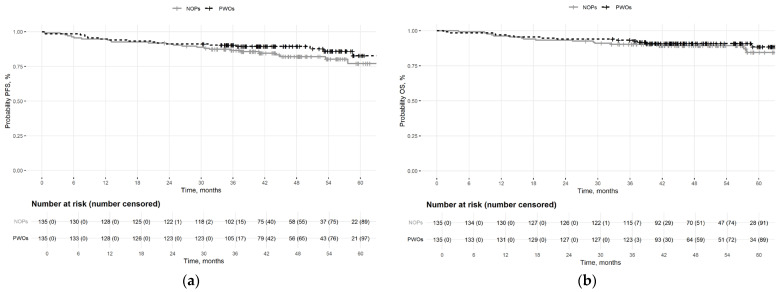
Kaplan–Meier curves. (**a**) Progression-free survival for patients with obesity (PWOS) (black) and non-obese patients (NOPs) (grey). (**b**) Overall survival for PWOs (black) and NOPs (grey).

With a median follow-up of 50 months (95% CI: 48–53), 41 events (15.2%) were recorded. Most were deaths (n = 30, 11.1%), including five (1.8%) occurring during NAC. Among the remaining deaths, three (1.1%) occurred due to distant progression during NAC, 22 (8.1%) due to distant recurrence, one due to ovarian cancer, and two of unknown cause. Ten patients remain alive with metastatic disease. More events occurred in the NOP group (n = 24) than in the PWO group (n = 17). Median PFS and OS were not reached. At 4 years, 86% of patients were event-free (89% PWOs vs. 82% NOPs), and 90% were alive (91% PWOs vs. 89% NOPs) (Figure 2).

## 4. Discussion

This study examined the prevalence of overweight and obesity among patients with high-risk early breast cancer receiving neoadjuvant chemotherapy at a tertiary cancer center. In our cohort, 27.7% of patients were classified as obese, nearly twice the prevalence reported in the general Portuguese population (15.9%) [1]. Overall, 58.9% were either overweight or obese at the first oncology visit. Similar ranges have been described in a Danish study of breast cancer patients undergoing neoadjuvant chemotherapy (24.7% obese; 32.2% overweight) [20], whereas lower rates were reported among Italian women with early node-negative breast cancer (19% obese; 25.4% overweight) [21].

In our cohort, patients with obesity were predominantly older, postmenopausal, and more likely to present with HR-positive/HER2-negative tumors, consistent with previously reported epidemiologic patterns in breast cancer [5]. Despite a similar age distribution, germline genetic testing was less frequently performed in patients with obesity than in non-obese patients. This may be explained by the higher prevalence of triple-negative disease among non-obese patients.

Multivariable logistic regression suggest that obesity constitutes an independent risk factor for chemotherapy-related toxicity, particularly treatment discontinuation, in Portuguese patients with early breast cancer. This association remained significant after adjustment for age, comorbidity burden, and carboplatin use, supporting the possibility of a direct biological effect of obesity on treatment tolerability. Although included in the composite endpoint, hospitalization and treatment discontinuation represent potentially more severe clinical consequences compared with treatment delays or dose reductions, and should be interpreted accordingly.

Age greater than 70 years was independently associated with dose reduction (*p* < 0.001), treatment discontinuation (*p* < 0.001), and hospitalization (*p* = 0.041). These findings are consistent with prior studies in early breast cancer [22]. Treatment delays were predominantly influenced by carboplatin use (*p* < 0.001), while the role of obesity in this context appears limited and inconclusive.

Although obesity was associated with an increased overall toxicity, particularly treatment discontinuation, its impact on pCR and survival outcomes could not be reliably evaluated given the limited number of patients and events, and the short follow-up period.

From a clinical perspective, it is important to distinguish between non-modifiable patient-related factors, such as age, and potentially modifiable contributors to treatment tolerance, including body composition and optimization of comorbid conditions.

Dose reductions occurred predominantly due to peripheral neuropathy in both groups. Treatment delays were mainly attributed to hematologic toxicity and infections in both groups. Hospitalizations showed a heterogeneous pattern. While treatment discontinuation was more strongly associated with obesity, peripheral neuropathy remained the leading cause of discontinuation in both cohorts. This observation may be explained by the limitation of BMI, which does not capture detailed body composition, such as fat distribution, lean mass, or muscle quality. These factors are important determinants of clinical outcomes and chemotherapy-related toxicity [23,24,25]. More recently, increasing attention has been directed toward body composition as a more accurate predictor of treatment-related toxicity risk. Indeed, higher fat mass and lower muscle mass have been associated with increased chemotherapy-related toxicities, independent of BMI [26,27]. In a meta-analysis evaluating chemotherapy-induced peripherical neuropathy, taxane-based therapies showed the strongest association in patients with elevated BMI, whereas cisplatin and anthracyclines have shown weaker or non-significant associations [28]. Evidence from multiple studies indicates that obesity is associated with treatment-related toxicities and clinically worse outcomes in patients with breast cancer [29,30]. Current guidelines do not recommend dose capping at initial chemotherapy prescription due to the risk of compromising treatment efficacy [8]; however, clear guidance on toxicity mitigation strategies in high-risk patients remains limited. A recent investigation suggests that tailoring paclitaxel dosing according to body composition—such as prolonging infusion duration—may reduce peripheral neuropathy while preserving adequate systemic exposure in patients with low muscle mass [31]. A meta-analysis of randomized controlled trials evaluating exercise performed before and/or during taxane-containing chemotherapy in women with breast cancer demonstrated reduced peripheral neuropathy symptoms and improved health-related quality of life compared with usual care, suggesting a potential biological role of exercise in modulating neuropathy persistence [32]. Exercise currently receives weak recommendations in the European Society for Medical Oncology (ESMO) guidelines for the prevention ([II, C]) and management ([II, B]) of neuropathy in at-risk populations [33]. Nutritional counseling, alone or combined with structured exercise programs during chemotherapy, appears safe and may mitigate selected treatment-related toxicities [34,35,36]. In addition, structured electronic patient-reported outcome monitoring enables earlier symptom detection and has been associated with reduced emergency visits and improved symptom control during systemic therapy [37]. These strategies were not evaluated in the present study. Future prospective studies should incorporate body composition measures to better identify patients at increased risk and explore supportive care interventions aimed at reducing treatment-related toxicities.

This study has inherent limitations, including its retrospective design and a relatively small number of participants, which may have reduced statistical power and increased the risk of bias. In addition, as the cohort was drawn from a single institution, the generalizability of our findings to broader populations may be limited. The short follow-up period and the small number of events precluded a reliable analysis of PFS and OS.

Although BMI is easily accessible and time-efficient, it does not reflect body composition or muscle quality, which are important determinants of clinical outcomes and chemotherapy-related toxicity [23,24,25,26,27]. Furthermore, the lack of longitudinal weight data is an additional limitation, as patients may have experienced weight gain or weight loss after diagnosis, potentially influencing outcomes. Finally, toxicity data were derived from clinician-documented records, and patient-reported outcomes were not systematically collected. Therefore, overall symptom burden may not be fully captured. However, the study focused specifically on events that resulted in treatment modification.

## 5. Conclusions

In conclusion, our findings provide insight into the magnitude of this clinical issue, revealing a substantial proportion of obesity among patients undergoing neoadjuvant chemotherapy. These patients may require closer monitoring for treatment-related toxicities, particularly treatment discontinuation due to peripheral neuropathy. Reliance on BMI alone limits the ability to fully assess body composition, and future studies incorporating body composition metrics are warranted to better refine toxicity risk and guide clinical decision-making.

## Figures and Tables

**Table 1:** Patient demographics for overall population.

Variable	Overall Cohort (N = 487)	Patients with Obesity (n = 135)	Non-Obese Selected Patients (n = 135)
Male sex, n (%)	1 (0.2)	0 (0.0)	1 (0.7)
Postmenopausal, n (%)	247 (50.7)	81 (60)	76 (56.3)
Median age (range) years	52 (20–82)	55 (31–79)	53 (20–79)
Age group, n (%)			
Age < 50 years	205 (42.1)	45 (33.3)	45 (33.3)
Age 50–70 years	230 (47.2)	75 (55.0)	75 (55.0)
Age > 70 years	52 (10.7)	15 (11.1)	15 (11.1)
Median BMI, kg/m^2^	26.3	32.6	24.1
BMI category, n (%)			
BMI < 18.5 kg/m^2^	14 (2.9)	0	3 (2.2)
BMI 18.5–24.9 kg/m^2^	186 (38.2)	0	75 (55.6)
BMI 25–29.9 kg/m^2^	152 (31.2)	0	57 (42.2)
BMI ≥ 30 kg/m^2^	135 (27.7)	135 (100)	0 (0.0)

**Table 3:** Significant toxicities.

Clinically Significant Toxicities	PWO	NOP
**Global** **n (%)**	**81 (60)**	**61 (45.2)**
**Dose reductions, n (%)**	**43 (31.8)**	**41 (30.4)**
From start	9	9
Fatigue	3	4
Hematologic	1	8
Febrile neutropenia	1	0
Peripheral neuropathy	16 (37.2)	13 (31.7)
Dermatologic	5	5
Liver function tests elevation	2	1
Diarrhea	3	
Nausea	2	
Acute kidney injury	0	1
Vertigo syndrome	1	0
**Treatment delays, n (%)**	**36 (26.6)**	**26 (19.3)**
Infection	16 (44)	6 (23)
Febrile neutropenia	2	2
Hematologic	15 (41.7)	14 (53.8)
Liver function tests elevation	1	0
Diarrhea	2	0
Pelvic fracture	0	1
Pulmonary embolism	0	2
Vertigo syndrome	0	1
**Hospitalizations, n (%)**	**14 (10.4)**	**7 (5.2)**
Infection	3	2
Febrile neutropenia	5 (35.7)	3 (42.8)
Pulmonary embolism	1	2
Pneumonitis	1	0
Hepatitis	1	0
Hypocalcemia	1	0
Neurological Ataxia and dysarthria/hemorrhagic stroke	2	0
**Treatment discontinuation, n (%)**	**29 (21.5)**	**19 (14.1)**
Reduced left ventricular ejection,	0	1
Infection	1	1
Febrile neutropenia	0	2
Peripheral neuropathy	15 (51.7)	8 (42.1)
Dermatologic	1	1
Pneumonitis	3	1
Hepatitis	1	0
Diarrhea	1	0
Pulmonary embolism	1	0
Decline in performance status	6 (20.7)	1 (5.3)
Disease progression	1	1
Death	2	3

**Table 4:** Multivariable logistic regression analysis of factors associated with global clinically significant toxicity.

	OR (95% CI)	*p*-Value
Obesity (yes vs. no)	1.83 (1.08–3.15)	0.027
Age 50–70 vs. <50	1.62 (0.92–2.88)	0.098
Age > 70 vs. <50	9.83 (3.18–37.83)	<0.001
Comorbidity burden: 1 vs. 0	1.05 (0.57–1.93)	0.880
Comorbidity burden: ≥2 vs. 0	1.55 (0.71–3.43)	0.269
Carboplatin use (yes vs. no)	2.33 (1.16–4.84)	0.020

OR, odds ratio; CI, confidence interval. Adjusted for age group, comorbidity burden, and carboplatin use.

**Table 5:** Multivariable logistic regression analysis of factors associated with dose reduction.

	OR (95% CI)	*p*-Value
Obesity (yes vs. no)	1.03 (0.58–1.83)	0.923
Age 50–70 vs. <50	1.62 (0.85–3.16)	0.151
Age > 70 vs. <50	8.21 (3.02–23.88)	<0.001
Comorbidity burden: 1 vs. 0	1.00 (0.51–1.95)	0.998
Comorbidity burden: ≥2 vs. 0	1.42 (0.64–3.13)	0.385
Carboplatin use (yes vs. no)	1.13 (0.50–2.39)	0.763

OR, odds ratio; CI, confidence interval. Adjusted for age group, comorbidity burden, and carboplatin use.

**Table 6:** Multivariable logistic regression analysis of factors associated with treatment delays.

	OR (95% CI)	*p*-Value
Obesity (yes vs. no)	1.64 (0.87–3.13)	0.130
Age 50–70 vs. <50	1.74 (0.85–3.70)	0.140
Age > 70 vs. <50	2.46 (0.78–7.58)	0.119
Comorbidity burden: 1 vs. 0	0.97 (0.45–2.06)	0.942
Comorbidity burden: ≥2 vs. 0	1.48 (0.61–3.51)	0.380
Carboplatin use (yes vs. no)	5.73 (2.69–12.46)	<0.001

OR, odds ratio; CI, confidence interval. Adjusted for age group, comorbidity burden, and carboplatin use.

**Table 7:** Multivariable logistic regression analysis of factors associated with treatment discontinuation.

	OR (95% CI)	*p*-Value
Obesity (yes vs. no)	2.30 (1.18–4.59)	0.016
Age 50–70 vs. <50	2.13 (0.98–4.97)	0.066
Age > 70 vs. <50	7.09 (2.34–22.51)	<0.001
Comorbidity burden: 1 vs. 0	0.60 (0.27–1.30)	0.207
Comorbidity burden: ≥2 vs. 0	0.45 (0.17–1.11)	0.092
Carboplatin use (yes vs. no)	0.36 (0.08–1.10)	0.110

OR, odds ratio; CI, confidence interval. Adjusted for age group, comorbidity burden, and carboplatin use.

**Table 8:** Multivariable logistic regression analysis of factors associated with hospitalization.

	OR (95% CI)	*p*-Value
Obesity (yes vs. no)	1.95 (1.74–5.527)	0.187
Age 50–70 vs. <50	2.72 (0.80–12.47)	0.140
Age > 70 vs. <50	5.61 (1.11–33.22)	0.041
Comorbidity burden: 1 vs. 0	2.10 (0.70–6.72)	0.193
Comorbidity burden: ≥2 vs. 0	1.16 (0.28–4.61)	0.830
Carboplatin use (yes vs. no)	1.25 (0.27–4.31)	0.750

OR, odds ratio; CI, confidence interval. Adjusted for age group, comorbidity burden, and carboplatin use.

**Table 2:** Clinical characteristics of final cohort study.

Variable	PWO (n = 135)	NOP (n = 135)
**Median age, years (range)**	55 (31–79)	53 (20–79)
**Median BMI, kg/m^2^ (range)**	32.6 (30.1–53.5)	24.1 (16.9–29.7)
**ECOG, n (%)**		
0	108 (80)	120 (88.9)
1	26 (12.3)	7 (5.2)
≥2	1 (0.7)	1 (0.7)
**Comorbidities, n (%)**	86 (63.7)	46 (34.1)
Hypertension	70 (51.8)	33 (24.4)
Diabetes mellitus	21 (15.6)	11 (8.2)
Dyslipidemia	41 (30.4)	18 (13.3)
Chronic kidney disease	0	1 (0.7)
Cerebrovascular accident	2 (1.5)	1 (0.7)
Myocardial infarction	2 (1.5)	3 (2.2)
**Germline genetic testing performed, n (%)**	59 (43.7)	82 (60.7)
Negative (among tested),	49 (83.1)	76 (92.7)
BRCA 1/2 (among tested)	4	3
PALB 2 (among tested)	1	1
CHECK 2 (among tested)	1	0
ATM (among tested)	2	1
**Breast cancer subtype, n (%)**		
HR-positive/HER2 negative	68 (50.4)	53 (39.3%)
HER2-positive	36 (26.7)	39 (28.9%)
Triple-negative	31 (22.9)	43 (31.8%)
**Clinical stage, n (%)**		
I	4 (3.0)	3 (2.2)
II	88 (65.2)	93 (68.9)
III	43 (31.8)	39 (28.9)
**Node-positive (N+), n (%)**	89 (65.9)	78 (57.8)
**Histological grade, n (%)**		
Not specified	1 (0.7)	1 (0.7)
1	3 (2.2)	2 (1.5)
2	77 (57.0)	67 (49.6)
3	54 (40.0)	65 (48.1)
**Treatment regimen, n (%)**		
Anthracyclines + taxanes	79 (58.5)	74 (54.8)
Anthracyclines + taxanes + anti-HER2	36 (26.7)	39 (28.9)
Anthracyclines + taxanes + carboplatin	20 (14.8)	22 (16.3)

OR, odds ratio; CI, confidence interval. Adjusted for age group, comorbidity burden, and carboplatin use.

## Data Availability

Data are available from the corresponding author upon reasonable request, subject to ethical and institutional approval.

## Abbreviations

The following abbreviations are used in this manuscript:

BC	Breast cancer
BMI	Body mass index
NAC	Neoadjuvant chemotherapy
NOPs	Non-obese patients
PWOs	Patients with obesity
OS	Overall survival
pCR	Pathologic complete response
PFS	Progression-free survival
